# Lipid composition of the cancer cell membrane

**DOI:** 10.1007/s10863-020-09846-4

**Published:** 2020-07-26

**Authors:** Wojciech Szlasa, Iga Zendran, Aleksandra Zalesińska, Mounir Tarek, Julita Kulbacka

**Affiliations:** 1 Faculty of Medicine, Wroclaw Medical University, Wrocław, Poland; 2 Université de Lorraine, CNRS, LPCT, F-54000 Nancy, France; 3 Department of Molecular and Cellular Biology, Faculty of Pharmacy, Wroclaw Medical University, Wrocław, Poland

**Keywords:** Lipid membrane, Membrane composition, Cancer cells

## Abstract

Cancer cell possesses numerous adaptations to resist the immune system response and chemotherapy. One of the most significant properties of the neoplastic cells is the altered lipid metabolism, and consequently, the abnormal cell membrane composition. Like in the case of phosphatidylcholine, these changes result in the modulation of certain enzymes and accumulation of energetic material, which could be used for a higher proliferation rate. The changes are so prominent, that some lipids, such as phosphatidylserines, could even be considered as the cancer biomarkers. Additionally, some changes of biophysical properties of cell membranes lead to the higher resistance to chemotherapy, and finally to the disturbances in signalling pathways. Namely, the increased levels of certain lipids, like for instance phosphatidylserine, lead to the attenuation of the immune system response. Also, changes in lipid saturation prevent the cells from demanding conditions of the microenvironment. Particularly interesting is the significance of cell membrane cholesterol content in the modulation of metastasis. This review paper discusses the roles of each lipid type in cancer physiology. The review combined theoretical data with clinical studies to show novel therapeutic options concerning the modulation of cell membranes in oncology.

## Introduction

Cells in the body differ from each other to maintain the proper functioning of the tissues and organs, which they build. The lipid profile of the plasma membrane is a distinctive cell property and might be considered as characteristic for a specific cell type (Pradas 2018). Alterations arise from particular cell functions and are directly caused by increased production of various lipid components (Pradas 2018). The discussed differences include the variability of the polar head groups, as well as of the non-polar fatty acids (FA) hydrocarbon chains (Casares 2019). The head groups in the outer leaflet of the lipid bilayer can differ in simple organic compounds added to the phosphate group (choline, ethanolamine, serine). Non-polar fatty acid moieties of the inner leaflet vary in hydrocarbon tail length and double bonds number or position (Rothman 1977).

The cell membrane is composed of lipids and proteins. It could either synthesise all the lipids, like hepatocytes or absorb the lipid fraction from lipoproteins circulating in the blood (Nagarajan 2019). The lipid content differs between tissues and depends on the physiological function of the cell. For instance, motile lymphocytes are characterised by low cholesterol level and high content of unsaturated lipids (Hoejholt et al., 2019). Conversely, non-motile tissues, that synthesise cholesterol are characterised by a high level of the steroid (Hoejholt et al., 2019). The lipid fraction of the plasmalemma could be divided into phosphatidic acid derivatives, glycolipids, sphingolipids and cholesterol (Fahy 2011). Phosphatidic acid could be substituted with inositol (phosphatidylinositol ~4 mol% of fibroblasts membrane), glycerol (phosphatidylglycerol ~0.5 mol%), serine (phosphatidylserine ~4 mol%), ethanolamine (phosphatidylethanolamine ~ 8 mol%) and choline (phosphatidylcholine ~20.25 mol%) (Fahy 2011; Hoejholt et al., 2019). Within phospholipids, the ether forms of the mentioned lipids can be distinguished, among which the most abundant are phosphatidylcholine ether (~0.25 mol%) and phosphatidylethanolamine ether (~7.75 mol%) (Fahy 2011; Hoejholt et al., 2019). Glycolipids are mostly composed of ceramide or phosphatidic acid conjugated with glycan group (Yu et al. 2010). Sphingolipids are the derivatives of sphingosine attached to choline/ethanolamine - forming sphingomyelin or glycans - forming cerebrosides (monosaccharide) or gangliosides (oligosaccharides) (Engelking and Engelking, 2015). Ceramide – sphingosine with fatty acid residue, is the apoptosis-inducing lipid signal (Van Blitterswijk et al. 2003). Cholesterol is the steroid compound, which content highly depends on the origin of the cell (Hoejholt et al., 2019; Pradas 2018). Namely, the motile cells, like lymphocytes are characterised by ~20 mol% plasma-cholesterol content and non-motile steroidogenic tissue cells by above 35 mol% content (Hoejholt et al., 2019). In general, cholesterol is responsible for the cell membrane rigidity and unsaturated phospholipids for the fluidity of the cell membrane (Gracià et al., 2010; Kakorin et al. 2005). Aside from the structural role, the lipids also provide the proper microenvironment for the membrane proteins to attach (Buschiazzo et al., 2013). Proteins could be either integral part of the membrane or could be anchored by the lipid chain to the lipid bilayer (GPI) (Brown and Waneck, 1992). There might be a distinguished group of proteins that selectively accumulate in the cholesterol and sphingomyelin-full microdomains of the membranes (lipid rafts), and proteins that localise in the non-raft region (Donatello et al., 2012). The proteins are organised next to each other, allowing their direct interactions (Sezgin 2017). Membrane proteins could be responsible for physical stabilisation of the membrane, acting as the receptors or being responsible for transport across the membrane – channels and transporters (Pike 2003; Podo et al., 2016). Some proteins are also involved in signal transduction or play an enzymatic role (Lane 2015; Stellacci et al. 2009). In most cases, interactions of the lipids with cholesterol or sphingomyelin in rafts is crucial for their proper functioning, thus the destabilisation of the rafts, lead to severe clinical complications (Björkholm 2014; Weiser 2014).

Cancer cell lipid composition differs from the non-malignant cell profile, but it also varies between malignancy types (Bernardes and Fialho, 2018; Casares 2019; Pakiet et al., 2019). Unfortunately, there is no specific lipid profile characteristic for cancer cells, that would differentiate them from non-malignant cells (Perrotti 2016). Moreover, cancer cell lipid composition may fluctuate in time depending on its physiological condition. For instance, cells preparing for metastasis, reduce membrane cholesterol content to increase the membrane fluidity and plasticity, which is essential for penetrating blood vessels (Zalba and ten Hagen, 2017). The disorganisation of lipids in the membrane can significantly disturb cell signalling (Lladó et al., 2014). Molecular dynamics studies showed that the loss of lipid asymmetry, observed in cancer cells, leads to a decrease in their permeability (Rivel et al. 2019).

In this review paper, we present a comprehensive overview of alterations in the lipid profile of the cell membrane of cancer in correlation with its biological significance. Membrane characteristics of various malignancies, based on most recent lipidomic profiling studies are discussed as well.

## Membrane phospholipids

### Phosphatidic acid (PA)

**Fig. 1.**
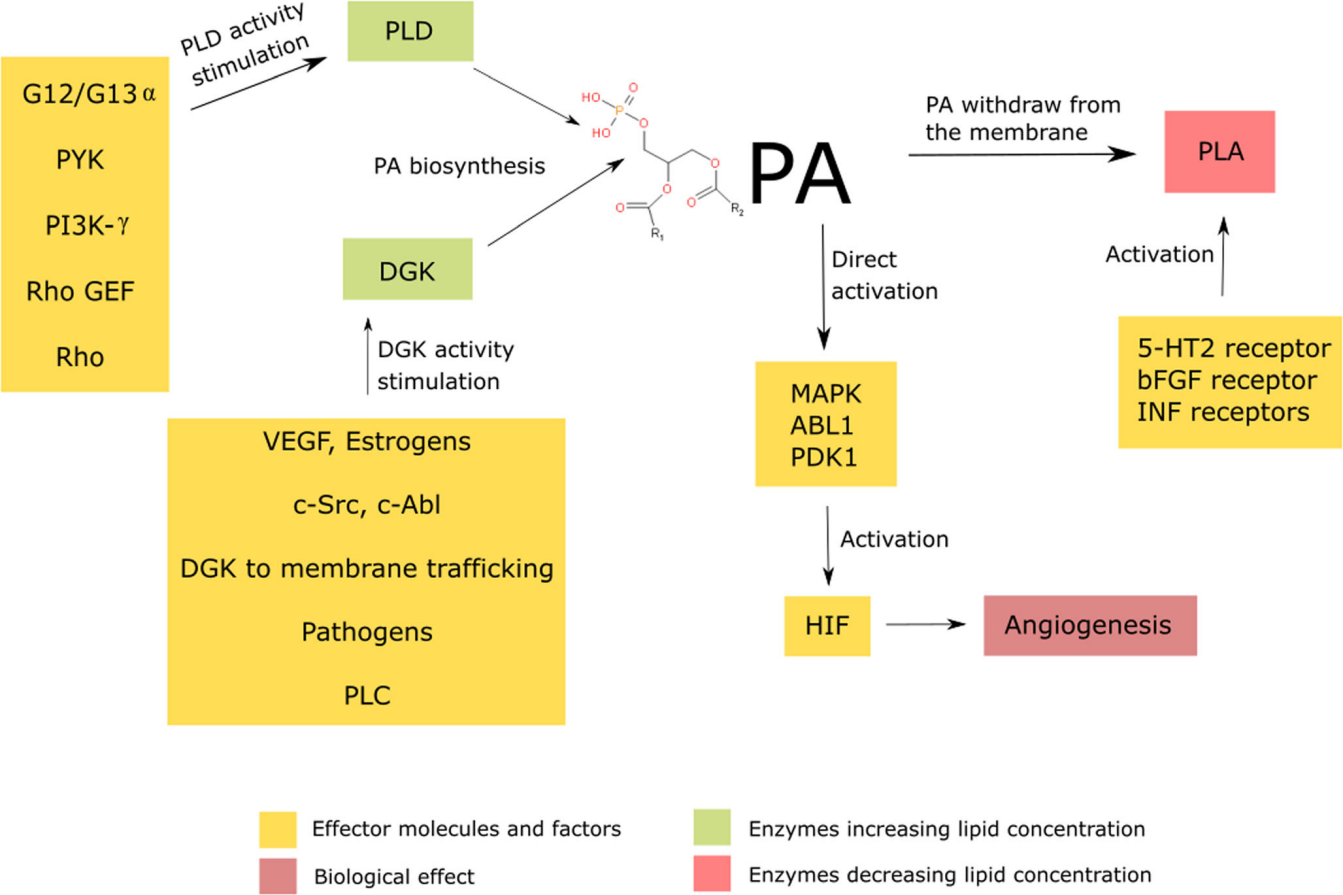
Phosphatidic acid (PA) biosynthesis is being stimulated by VEGF, estrogens, c-Src and c-Abl. Pathogens, PLC and DGK trafficking towards cell membrane increase the PA biosynthesis. The lipid directly activates MAPK, ABL1 and PDK1 kinases, leading to the HIF activation and thus neovascularization. Conversely, factors stimulating PLD, like PYK, PI3K-gamma, G12/13 alpha subunits activation, Pho GEF and Rho, lead to the increased activity of PLD, which catalyzes the PA degradation; DGK - diacylglycerol kinase, PLD – phospholipase D, VEGF – vascular endothelial growth factor, c-Src - Proto-oncogene tyrosine-protein kinase Src, c-Abl - Abl protein-tyrosine kinase, PLC – phospholipase C, HIF - hypoxia-inducible factor, MAPK - mitogen-activated protein kinases, ABL1 - Abelson murine leukaemia viral oncogene homolog 1, PDK1 - Phosphoinositide-dependent kinase-1, G12/G13α – α subunits of heterotrimeric G proteins, PYK - pyruvate kinase, PI3K-γ - phosphoinositide 3-kinase γ, Rho - Ras homologous protein factor, Rho GEF – Rho guanine exchanging factor;

Phosphatidic acid consists of diacylglycerol backbone bound to a phosphate group (Putta et al., 2016). Under physiological conditions, it accumulates on the cytoplasmic side of the plasma membrane due to the negative charge of the hydrophilic head group (Van Meer et al., 2008). Hydrophobic moieties are represented mostly by oleic, stearic and palmitic acids (Zech et al., 2009). The second binding position on the glycerol molecule is commonly occupied by an unsaturated fatty acid hydrocarbon chain (Zech et al., 2009). The content of phosphatidic acid physiologically does not exceed 1 mol% of membrane lipids (Zech et al., 2009). Nevertheless, due to its negatively-charged phosphate group, phosphatic acid plays a significant physiological function. Alternations in the level of membrane phosphatidic acid are regulated by changes in its metabolism (Fig. 1). Diacylglycerol kinase (DGK) and phospholipase D (PLD) are crucial enzymes involved in PA biosynthesis pathways (*Liscovitch et al.,* 2000; *Matsubara et al.,* 2006). Conversely, the degradation of PA is indirectly caused by phospholipase A (PLA) (Brindley 1996, 1998). DGK is regulated in a hormonal way. The stimulation by vascular endothelial growth factor (VEGF) leads to the activation of Src protein-tyrosine kinase (c-Scr) and Abl protein-tyrosine kinase (c-Abl), leading to the enhance in DGK activity (Baldanzi et al. 2008; Baldanzi et al., 2004; Matsubara et al., 2012). This promotes DGK trafficking to the plasma membrane, which eventually induces PA biosynthesis (Purow 2015). DGK may be activated by pathogen-induced enhanced phospholipase (PLC) activity as well (Stith 2015). PA biosynthesis is also catalysed by PLD which activity is G-protein-dependent (Brandenburg 2014). The G-proteins’ β and γ subunits (Gβγ) function as negative regulators of PLD activity, whereas G12/G13 proteins’ α subunits promote the activity via several other factors, such as pyruvate kinase (Pyk), phosphoinositide-3-kinase-γ (PI3K-γ), Rho guanine nucleotide-exchange factor (Rho GEF) and Rho factor (Hess et al. 1997; Lucas et al. 2002; *Mariggiò et al.,* 2006; *Preininger et al.,* 2006). Changes in the activity of the above-mentioned proteins are associated with carcinogenesis. Interestingly, aside from cancer, the process of PLD activation is also involved in physiological processes, such as wound healing (Arun et al. 2013).

PLA is responsible for the conversion of PA into lysophospholipid, which results in its withdrawal from the cell membrane to the cytoplasm. PLA activity highly depends on the activity of membrane hormone receptors, such as 5-HT2, bFGF and INF receptors (Goddard 1992; Kurrasch-Orbaugh 2003; Wu et al., 1994). bFGF activates PLA2 indirectly, by enhancing the release of PLA2-activating protein (Goddard 1992). With the stimulation of INF-α/β/γ and 5-HT2R, PLA activity increases as well (Kurrasch-Orbaugh 2003; Wu et al., 1994). Conversely, PLA2 deactivates in response to glutamine and oxidants (*Huang et al.,* 2020; Lee 2015).

Elevated levels of PA are related to a series of changes to cancer cell metabolism. Primarily, it activates kinases, such as mitogen-activated protein kinase (MAPK), ABL tyrosine kinase 1 (ABL1) or 3-phosphoinositide-dependent protein kinase-1 (PDK1), implicated in intracellular stress signalling pathways (Lee et al. 2001; Plattner et al. 1999; Putta 2016). The aforementioned enzymes are also associated with cancer initiation and progression (Papageorgis et al. 2011; Papandreou et al. 2011; Reynaert et al. 1992). Moreover, the elevated level of PA enhances hypoxia-inducible factor 1-alpha (HIF1A) transcription which promotes angiogenesis and cancer cell proliferation (Han et al. 2014).

### Phosphatidylinositol (PI)

Phosphatidylinositol (PI) family is a heterogeneous group of membrane lipids that differ from each other with regard to fatty acid moieties composition and the number of phosphate groups attached to polar myoinositol head group (Yeagle 2016). Phosphoinositides are the phosphorylated forms of phosphatidylinositol (Falkenburger et al., 2010). It is possible to distinguish phosphatidylinositol mono- and bisphosphates phosphorylated on the C3, C4 or C5 positions of the inositol ring, as well as triphosphate with phosphate groups in all three of the positions (Yeagle 2016). Phosphorylation is possible due to the specific activity of phosphatidylinositol phosphate kinases (PIPK) (Muftuoglu et al. 2016; Rajala and Anderson 2010). Conversely, dephosphorylation is catalysed by specific phosphatases (Hsu and Mao, 2015).

Plasma membrane level of PI is volatile and fluctuates between 5 and 12 mol% with regard to HT-29 colon cancer cell line and Jurkat cell line, respectively (*Hoejholt et al.,* 2019; *Zech et al.,* 2009). Under physiological conditions, phosphoinositides are present in the outer leaflet of the membrane (*Van Meer et al.,* 2008). Most phosphoinositides contain stearic or oleic acid in the sn-1 carbon; arachidonic or oleic acid is bound by sn-2 carbon atom (*Zech et al.,* 2009). However, as a result of a high diversity of the phosphoinositol family and variety of their metabolic functions, it is impossible to fully comprehend the contribution of phosphoinositides to the lipid membrane profile.

Each of the phosphoinositides exhibits specific metabolic activity and affects different cell signalling pathways. Phosphatidylinositol-3-phosphate recruits proteins involved in membrane trafficking (*Nascimbeni et al.,* 2017). Phosphatidylinositol-4-phosphate is an important component of the membrane of the Golgi apparatus, recruiting proteins for the transport to the cell membrane (*Hammond et al.,* 2014). Conversely, phosphatidylinositol 5-phosphate participates in proliferation control by modulating both histones acetylates and deacetylases activity, as well as p53 acetylation (*Poli et al.,* 2019). The activity of the enzyme is performed via the inhibitor of growth 2 (ING2) (*Poli et al.,* 2019). Curiously, phosphatidylinositol monophosphates are involved in membrane curvature regulation (*Gallop et al.,* 2013).

Phosphatidylinositol bisphosphates (in particular phosphatidylinositol 4,5-bisphosphate) function as secondary messengers in many signalling pathways (*Falkenburger et al.,* 2010). Besides, phosphatidylinositol 4,5-bisphosphate binds proteins responsible for actin filaments organization, as well as stabilises G protein-coupled receptors, enhancing the effectiveness of various signalling pathways (*C. Y.* *Wu et al.,* 2014; *Yen et al.,* 2018). Additionally, phosphatidylinositol 4,5-bisphosphate promotes the membrane recruitment of G protein-coupled receptor kinase 2 (GRK2) - one of the G protein effector molecules (*Pitcher et al.,* 1996).

Phosphatidylinositol bisphosphates are not located only in the plasma membrane, but also in the endomembrane system. Phosphatidylinositol 3,5-bisphosphate may be found in the intracellular membranes, where it forms vacuole and alveoli, as well as participates in exocytosis (*Li et al.,* 2013). Therefore, the lipid is of particular importance for actively secreting cancer cells (*King et al.,* 2012). Considering the above, cancer cell secretion could be inhibited by targeting the phosphatidylinositol 3,5-bisphosphate biosynthesis pathway (*Thapa et al.,* 2016). Phosphatidylinositol bisphosphates are involved in ion channels regulation as well. (*Mccartney et al.,* 2014). By affecting the potassium channels, the resting membrane potential is modulated, serving as a proliferative signal. It is frequently found in cancer cells (*Yang and Brackenbury,* 2013).

Phosphatidylinositol (3,4,5)-trisphosphate accounts for numerous cellular signalling pathways activation, i.e. protein kinase B (AKT) pathway, therefore contributing to cell growth and survival regulation (*Manna and Jain,* 2015). Like other phosphoinositides, phosphatidylinositol (3,4,5)-trisphosphate participates in the recruitment of proteins with pleckstrin homology domain (PH domain) (*Milburn et al.,* 2003). These include a large number of proliferation-related kinases, such as AKT, PDK1 or Bruton's tyrosine kinase (BTK) (*Manna and Jain,* 2015).

One of the most significant functions of phosphatidylinositol phosphates is the formation of GPI-anchor, thus allowing the motile protein-membrane interactions (*Brown and Waneck,* 1992). Phosphatidylinositol enables attachment of freely floating proteins to the lipid bilayer of the membrane through hydrophobic interactions within the nonpolar membrane core (*McLaughlin and Murray,* 2005).

**Fig. 2.**
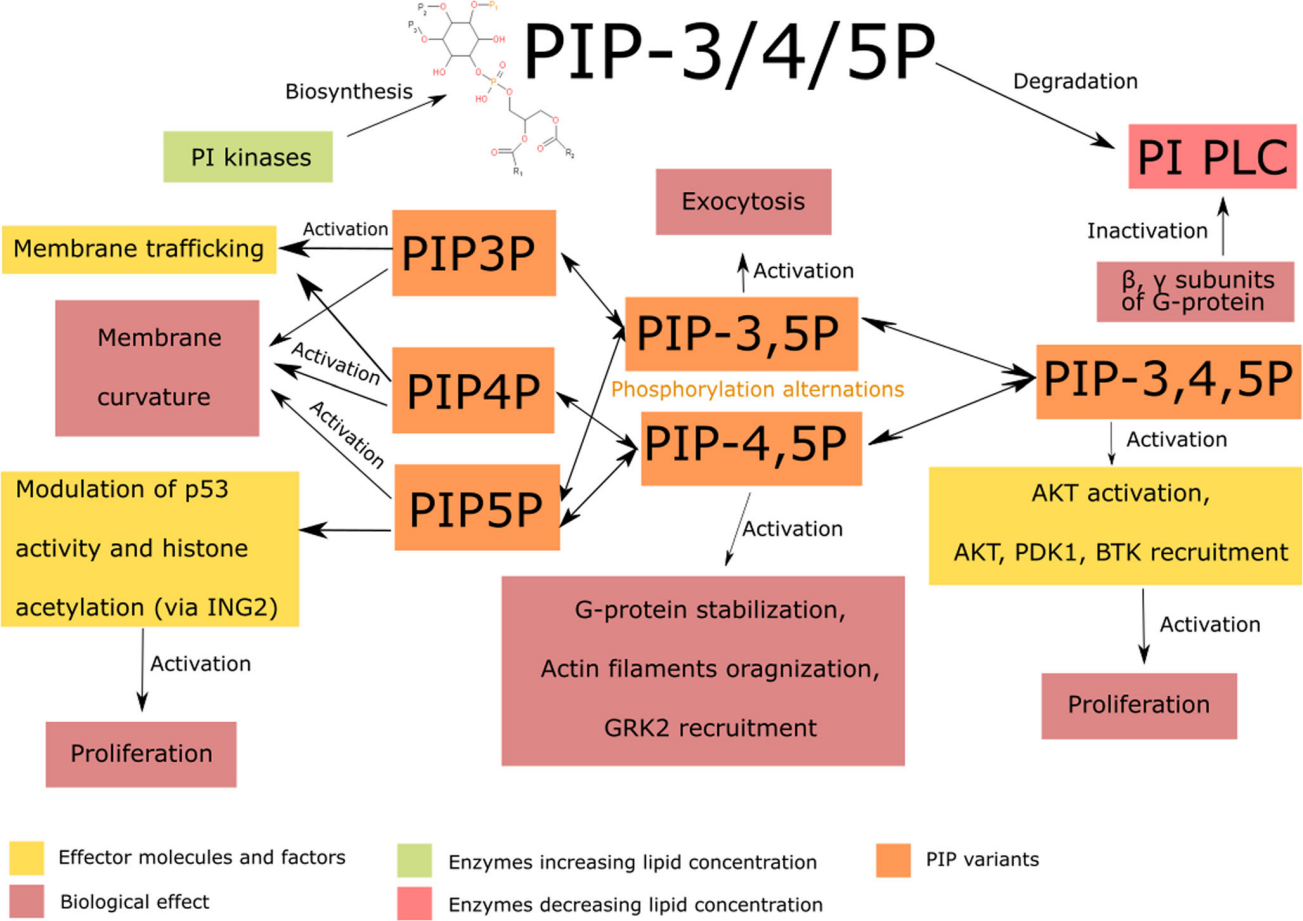
Phosphoinositides (PI) metabolism and action scheme. Diphorylated phosphatidylinositol forms are responsible for cancer-related microsomes exocytosis, G-protein stabilization and constant activation, actin filaments prometastatic organization and GRK2 mediated proliferator signal transduction. Various mono-phosphorylated forms of phosphatidylinositol are responsible for membrane curvature modulation or induction of proliferation; PIP-3/4/5P - phosphatidylinositol 3/4/5-phosphate, PIP-3,5P - phosphatidylinositol 3,5-diphosphate, PIP-4,5P - phosphatidylinositol 4,5-diphosphate, PIP-3,4,5P - phosphatidylinositol 3,4,5-trisphosphate, ING2 - inhibitor of growth protein 2, p53 - transformation-related protein 53, AKT – protein kinase B, PDK1 - protein 3-phosphoinositide-dependent protein kinase-1, BTK - Bruton's tyrosine kinase, GRK2 - G-protein-coupled receptor kinase 2; PI PLC - PI specific phospholipase C.

Phosphoinositides biosynthesis impairment is caused by specific kinase dysfunction. Contrarily, phosphatidylinositol accumulation occurs when PI specific phospholipase C (PI PLC) remains inactive or mutated (*Cocco et al.,* 2006). β and γ subunits of G-protein decreases the activity of PI PLC (fig. 2) (Rhee 2001).

### Phosphatidylglycerol (PG)

Phosphatidylglycerol structure consists of phosphatidic acid bound to glycerol substituent (*Yeagle* 2016). The lipid is an anionic intermediate of the cardiolipin biosynthesis pathway (*Yeagle* 2016). Although phosphatidylglycerol comprises only about 1 mol% of the membrane phospholipids, it remains an important component of the cytosolic side of the plasma membrane due to its ionic properties (*Zech et al.,* 2009). Oleic and palmitic acid moieties are the most common glycerol substituents for phosphatidylglycerol (*Zech et al.,* 2009).

**Fig. 3.**
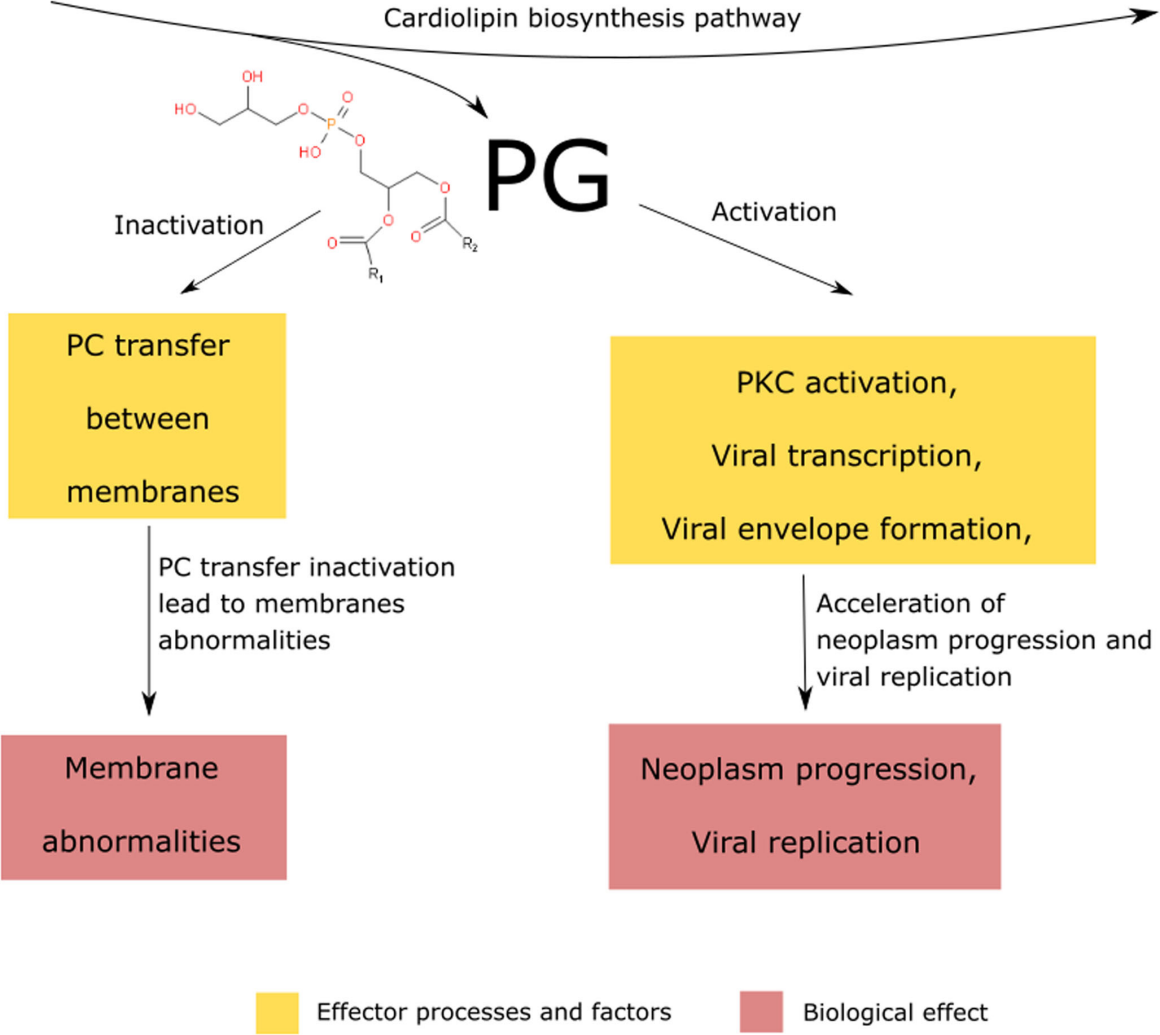
Phosphatidylglycerol (PG) is being synthesized as the intermediate metabolite in cardiolipin biosynthesis pathway. The lipid inhibits PC transfer between membranes, leading to the cancerous membrane abnormalities. PG activatess PKC, viral transcription and envelope formation, leading to the neoplasm progression and increased ratio of viral replication; PC - phosphatidylcholine, PKC – protein kinase C;

Certain studies have demonstrated that phosphatidylglycerol accounts for protein kinase C (PKC) activation and that it is involved in viral transcription (*Bailey et al.,* 2017; *Hirai et al.,* 1992; *Murray and Fields,* 1998). Moreover, phosphatidylglycerol participates in viral envelope formation (*Sands and Lowlicht,* 1976). At the tissue level, phosphatidylglycerol can inhibit phosphatidylcholine transfer between membranes, which results in membrane structure and function irregularities (*Wirtz et al.,* 1976). The importance of this compound during viral infections may suggest that its abnormal levels could be observed in virus-associated cancers such as cervical cancer (*Preetha et al.,* 2005). These fluctuations may lead to an increase in viral replication effectiveness and cell neoplasia (Fig. 3).

### Phosphatidylserine (PS)

Phosphatidylserine under physiological pH is defined as negatively charged lipid, present in the intracellular layer of the cell membrane (Yeagle 2016). Its volume in the cell membrane is estimated at around 6 mol% (*Zech et al.,* 2009). Its degradation by decarboxylation merges with the biosynthesis process of phosphatidylethanolamine, which occurs in large quantities in the plasma membrane (*Dawaliby et al.,* 2016). Like PE, PS is mostly composed of palmitic, oleic and stearic acids attached at the C1’ glycerol position and residues of oleic acid in C2' position (*Zech et al.,* 2009).

**Fig. 4.**
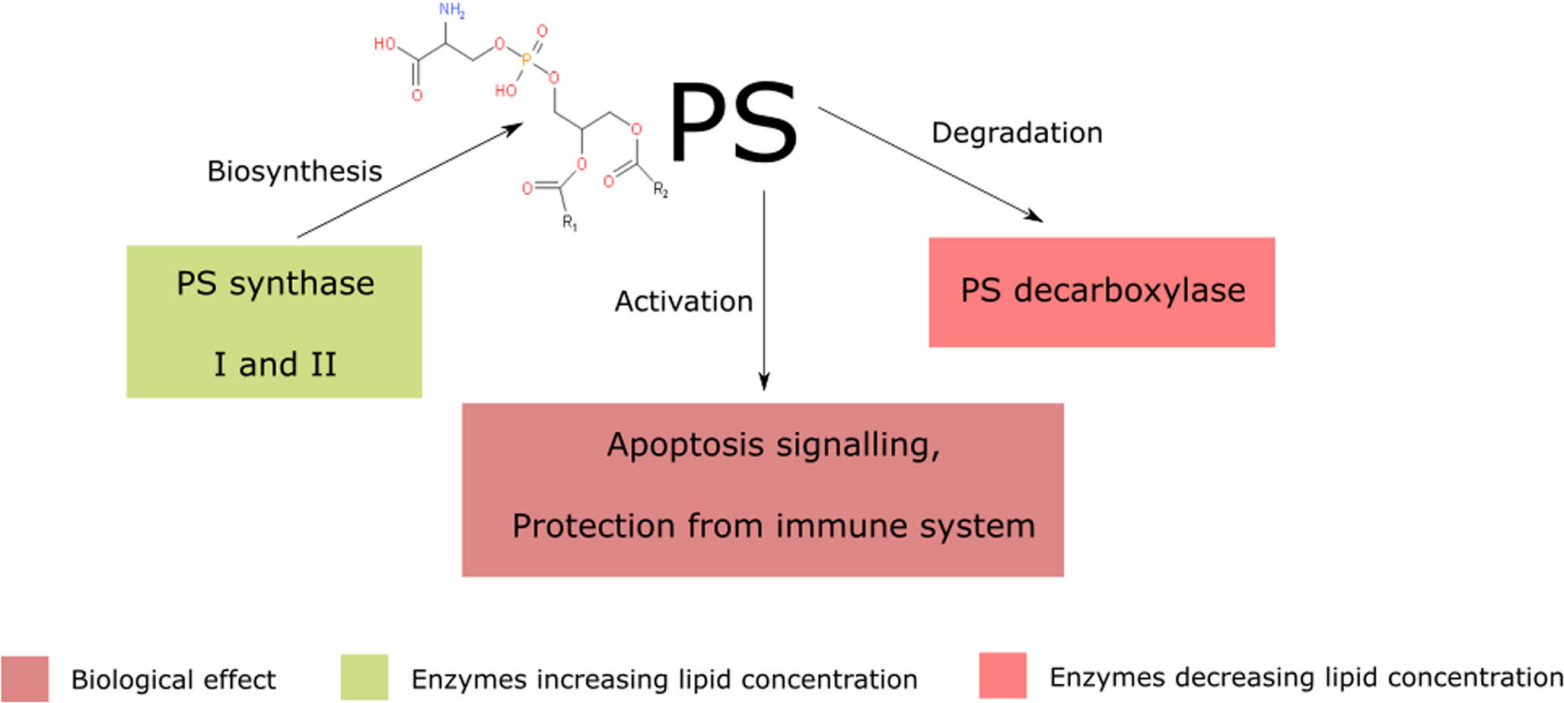
Phosphatidylserine (PS) biosynthesis is being catalyzed by PS synthase I or II. The degradation is catalyzed by PD decarboxylase. The increased PS content in the cell membrane leads to cancer cell protection from the immune response. However, the increase in PS in the outer membrane of the cell leads to the escalation of apoptosis signaling.

The biosynthesis of phosphatidylserine is associated with the activity of two phosphatidylserine synthase isoforms (PSS I and II) (*Stone and Vance,* 2000). Those catalyse choline to serine substitution in phosphatidylcholine and ethanolamine to serine substitution in phosphatidylethanolamine, respectively (*Arikketh et al.,* 2008; *Kuge & Nishijima* 1997; *Kuge et al.,* 1997). The process of phosphatidylserine degradation, however, takes place thanks to the phosphatidylserine decarboxylase which catalyzes its decomposition to phosphatidylethanolamine (Fig. 4) (*Vance and Steenbergen,* 2005).

The apoptosis signalling was considered to be the major cellular role of phosphatidylserine, however, nowadays, some authors prove that PS does not induce apoptotic but rather necroptotic cell death type (*Shlomovitz et al.,* 2019). This happens when the phosphatidylserine passes to the outer layer of the cell membrane due to the activity of an enzymatic protein called calcium ion-dependent flippase (Segawa and Nagata 2015). Physiologically, during apoptosis, phosphatidylserine is exposed on the cell surface, while in cancer cells, the process is present also during cellular oxidative stress (*Belzile et al.,* 2018). Due to this, phosphatidylserine excess may be considered as a tumour marker (*Sharma and Kanwar,* 2018).

Phosphatidylserine is exposed on the surface of cancer cells to avoid the autoimmune response of the organism (*Birge et al.,* 2016). It protects the cancer microenvironment from NK cells and other cytotoxic immune cells (*Lankry et al.,* 2013). Thus, cancer cells are no longer recognised as a threat to the immune system. Interestingly, numerous intracellular bacteria and viruses can induce similar effects, suggesting the convergent evolution of pathogens and cancers (*Birge et al.,* 2016). Currently, an attempt is made to target phosphatidylserine excessively exposing cells in cancer therapy (*Belzile et al.,* 2018; *Budha et al.,* 2018).

### Phosphatidylethanolamine (PE)

Phosphatidylethanolamine is the second most common (15-25 mol%) membrane phospholipid, occurring in ester or ether form (*Zech et al.,* 2009). The ether fraction prevails over the ester fraction under physiological conditions (*Zech et al.,* 2009). However, cells at a higher risk of disintegration of the plasma membrane tend to exhibit increased ether to ester ratio due to the greater thickness and denser packing of plasmalogens within the membranes (*Rog and Koivuniemi,* 2016). Due to its wedge shape, phosphatidylethanolamine is responsible for plasma membrane curvature modulation (*Lladó et al.,* 2014). Although phosphatidylethanolamine is present on both sides of the plasma membrane, it exhibits the greatest concentration in the cytosolic leaflet of the lipid bilayer (*Yeagle* 2016). Interestingly, in numerous cancer cell types, phosphatidylethanolamine distribution is reversed with a preference for the outer leaflet of the plasma membrane (*Liu et al.,* 2017a.

Chemical analysis shows that palmitic, stearic and oleic acid chains are the most common fatty acids residues in phosphatidylethanolamine structure (*Zech et al.,* 2009). Phosphatidylethanolamine ester is a product of phosphatidylcholine demethylation by phosphatidylcholine N-methyltransferase and phosphatidylserine decarboxylation by phosphatidylserine decarboxylase (*Bleijerveld et al.,* 2007). Alternatively, it could be synthesised via cytidine diphosphate-ethanolamine pathway, using ethanolamine as a substrate (*Bleijerveld et al.,* 2007). Conversely, the ether form is synthesised by the attachment of 1,2-unsaturated fatty acid residue at the phosphatidic acid C2 position. Afterwards, the fatty acid moiety is being detached and replaced by ethanolamine (*Honsho et al.,* 2017).

Phosphatidylethanolamine (PE) is involved in various plasma membrane processes. First of all, phosphatidylethanolamine acts as a lipid chaperone that assists in the folding of membrane proteins (*Bogdanov and Dowhan,* 1998). Thus, its lack would result in the excess in unfolded proteins, responsible for the metabolic shift towards cancerous phenotype (*Beloribi-Djefaflia et al.,* 2016; *Pustylnikov et al.,* 2018). Accordingly, lipid profile abnormalities may affect membrane protein performance in cell signalling pathways, resulting in metabolism dysregulation and impaired response to extracellular signals (*Kitajka et al.,* 2002). PE has quite remarkable property which is the ability to promote protein conversion towards the toxic conformation (*Supattapone* 2012). Excessive accumulation of misfolded proteins triggers chronic endoplasmic reticulum (ER) stress, which may lead to cancer development (*Patel and Witt,* 2017).

Additionally, phosphatidylethanolamine acts as a positive regulator of autophagy which has a significant, positive influence on cell condition by preventing it from accumulating abnormal organelles (*Rockenfeller et al.,* 2015). Malfunctioning organelles accumulation and uncontrolled degradation are the underlying cause of carcinogenesis (*Beloribi-Djefaflia et al.,* 2016).

Phosphatidylethanolamine exerts its intracellular biological functions via the phosphatidylethanolamine-binding protein (PEBP) (*Wang et al.,* 2004). PEBP acts as an inhibitor of serine proteases, negatively regulating the apoptotic process (*Rockenfeller et al.,* 2015). Furthermore, increased phosphatidylethanolamine levels were observed during laboratory-induced ER stress (*Shyu et al.,* 2019).

**Fig. 5:**
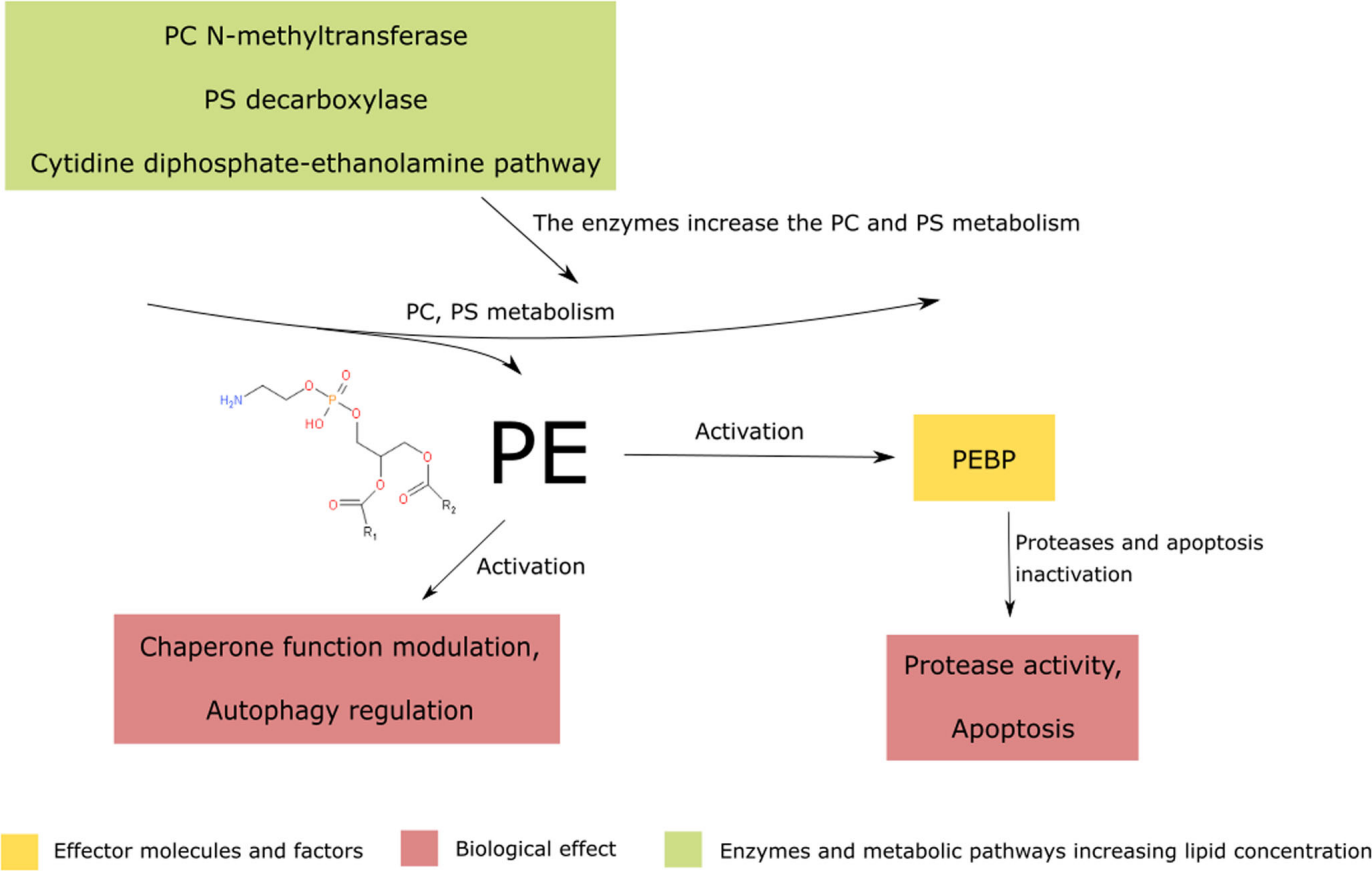
Phosphatidylethanolamine (PE) is being synthesized by the induction of PC N-methyltransferase, PS decarboxylase and CDP-ethanolamine pathway. The increased PE content in cell membrane leads to the activation of PEBP, which desensitizes the cell from the proapoptotic signals. Conversely, PE directly modulates the chaperone functions of the membrane-associated proteins. PC - phosphatidylcholine, PS - phosphatidylserine, PEBP – phosphatidylethanolamine binding protein.

As a part of the glycosylphosphatidylinositol (GPI) anchor, phosphatidylethanolamine is responsible for binding proteins to the membrane (*Menon et al.,* 1993; *Signorell et al.,* 2008). In this process, phosphatidylethanolamine is the donor of the phosphoethanolamine group that is attached either to the C-terminus of the protein or to the hydroxyl groups of the glycan-core mannose (*Patel and Witt,* 2017). The impairment of GPI-anchor biosynthesis results in improper stabilisation of the proteins in the membrane area (*Krawitz et al.,* 2013). Consequently, intracellular signalling pathways, biosynthesis and degradation reactions, as well as cell communication, may all be affected, leading to the cellular dysregulation and carcinogenesis (Fig. 5).

Unsaturated bonds in the hydrocarbon chains of phosphatidylethanolamine are susceptible to ROS-mediated oxidation (*Iuchi et al.,* 2019). The process is tightly connected with ferroptosis - a type of cell death, induced by the accumulation of lipid peroxides in the membrane (*Conrad et al.,* 2018). This results in regulated cell death, that lacks characteristic features of apoptosis, such as caspase activation (*Du et al.,* 2020; *Xu et al.,* 2019). Ferroptosis is thought to be the only type of the cell death from which cancer cells cannot escape, thus the researchers try to introduce it to cancer therapy (*Bebber et al.,* 2020; *Xu et al.,* 2019).

### Phosphatidylcholine (PC)

Phosphatidylcholine is a neutral membrane lipid, present in both ester and ether forms, the same as phosphatidylglycerol (*Yeagle* 2016). Although phosphatidylcholine is primarily a neutral lipid, it can also be referred to as a zwitterion, as it consists of a positively charged choline head group and a negatively charged phosphate group substituent (*Yeagle* 2016). Phosphatidylcholine is the most abundant compound of the lipid bilayer (*Zech et al.,* 2009). In comparison to phosphatidylethanolamine, it is mostly present in the outer leaflet of the plasma membrane and higher ester/ether form ratio (*Zech et al.,* 2009).

Considering common biosynthesis pathways, fatty acid residues of phosphatidylcholine are similar to those of phosphatidylethanolamine and phosphatidylserine (*Yeagle* 2016). Phosphatidylcholine is biosynthesised in the Kennedy pathway or phosphatidylethanolamine N-methyltransferase pathway (*Gibellini and Smith,* 2010). Impairment of phosphatidylcholine biosynthesis causes its membrane level to decrease simultaneously with an increase in phosphatidylethanolamine accumulation.

**Fig. 6.**
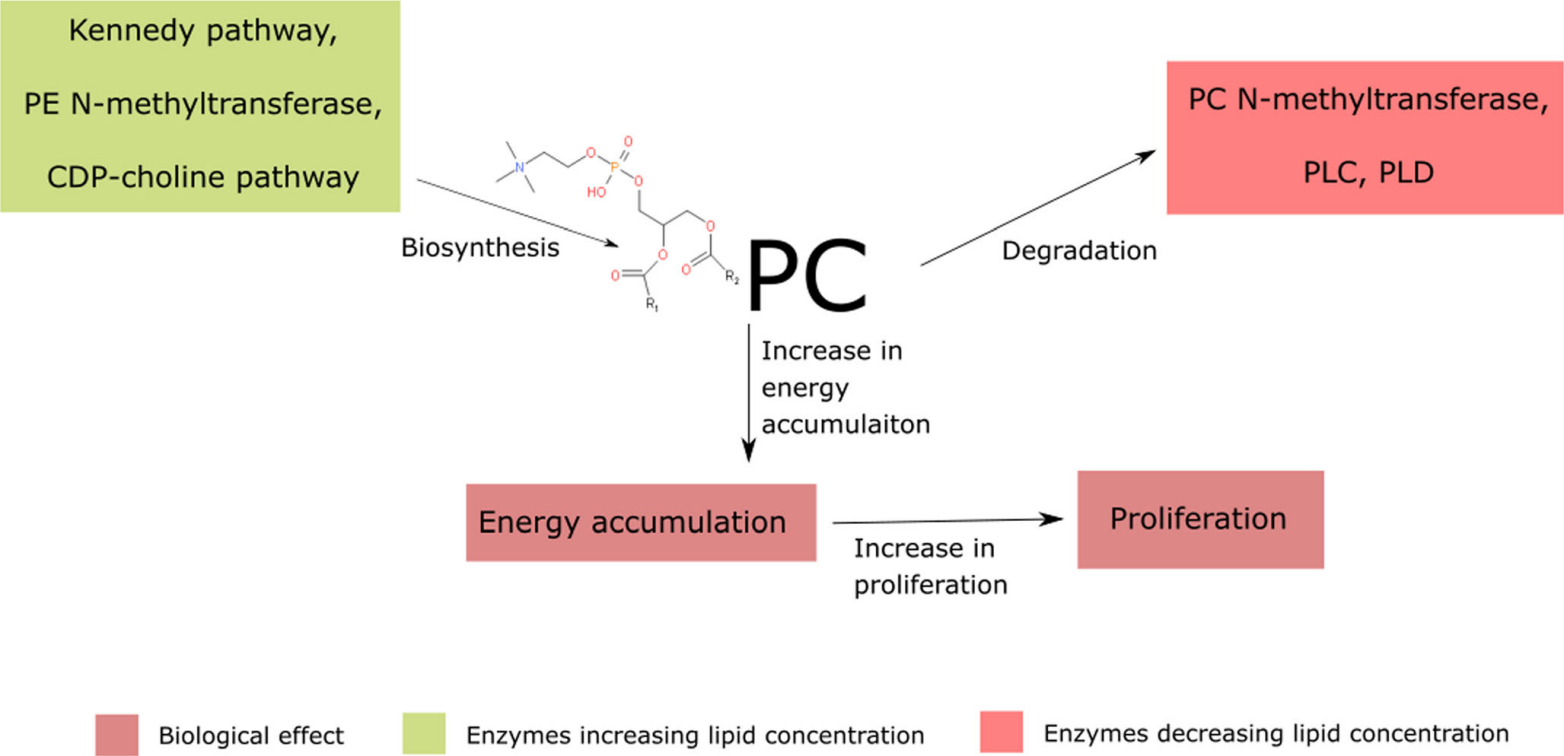
Phosphatidylcholine (PC) content in the cell membrane increases by the induction of the Kennedy pathway, PE N-methyltransferase and CDP-choline pathway. Conversely, the induction of PC N-methyltransferase, PLC and PLD leads to PC degradation. PC is the easily accessible energy for the cell which could be used for cancerous proliferation; PE - phosphatidylethanolamine, PLC - phosphatidylcholine, PLD – phospholipase D, CDP - cytidine diphosphate;

A wide range of cancers exhibits phosphatidylcholine metabolism alterations, caused mainly by choline kinase alpha (CHKA), phospholipase C (PLC) or phospholipase D (PLD) enhanced activity (*Hu et al.,* 2016). CHKA is responsible for the increase in phosphorylated choline level, serving as a substrate for cytidine diphosphate (CDP)-choline phosphocholine transferase in the CDP-choline pathway (*Arlauckas et al.,* 2016; *Sola-Leyva et al.,* 2019). Conversely, PLC catalyses the degradation of phosphatidylcholine with the release of diacylglycerol (DAG), promoting cellular anabolism via protein kinase A (PKA) (Oude Weernink et al. 2007; *Siso-Nadal et al.,* 2009). These metabolic shifts lead to the accumulation of energy-rich molecules, providing sources for cell proliferation. Carcinomas, such as breast cancer overexpress the above-mentioned enzymes (Fig. 6) (*Podo et al.,* 2016).

### Lysophosphatidylcholine and lysophosphatidylethanolamine

Lysophospholipids are phospholipids without an acyl chain at the C2 carbon position. Although lysophospholipids are nearly detectable in the plasma membrane, the lipids are involved in maintaining the proper functioning of the bilayer (*Van Meer et al.,* 2008). The anionic compounds possess entirely different properties from the fully acylated equivalents. Lysophospholipids biosynthesis is mediated by enhanced activity of phospholipase A2 (PLA2), while their degradation is catalysed by other phospholipases, like PLC, PLD or PLA1 (phospholipase A1). The elevation of lysophosphatidylethanolamine plasma levels is triggered by the plasma PLA2 (*Murakami et al.,* 2015). PLA2 activity stimulation results in lysophosphatidylipids production, leading to the improper membrane curvature and thus its destabilisation (*Henriksen et al.,* 2010). Consequently, plasma membrane leakage may arise. Moreover, in this state, the membrane becomes more susceptible to membrane fusion, making the lysolipids responsible for cell exocytic capacity (*Haraszti et al.,* 2016; *Hoen et al.,* 2016; *Leung et al.,* 1998). The increase in lysophospholipid content is responsible for cancer growth, proliferation, and angiogenesis presumably by enhancing the extracellular phospholipids delivered to the tumour (*Yang et al.,* 2018). Curiously, the elevated level of the lysophospholipid in the tumour microenvironment was proved to be a useful biomarker of ovarian cancer, which could become a potent anti-cancer target in the future (*Y.* *Xu* 2018). Presented properties explain the minor contribution of lysophospholipids to the membrane lipid profile. To prevent disintegration, the cell can incorporate the lacking fatty acid chains by a specific sn-2-acyltransferase or relocate the lysophospholipids from the plasma membrane to the cytosol (*Hishikawa et al.,* 2008; *Shindou et al.,* 2009).

### Membrane cholesterol

**Figure 7.**
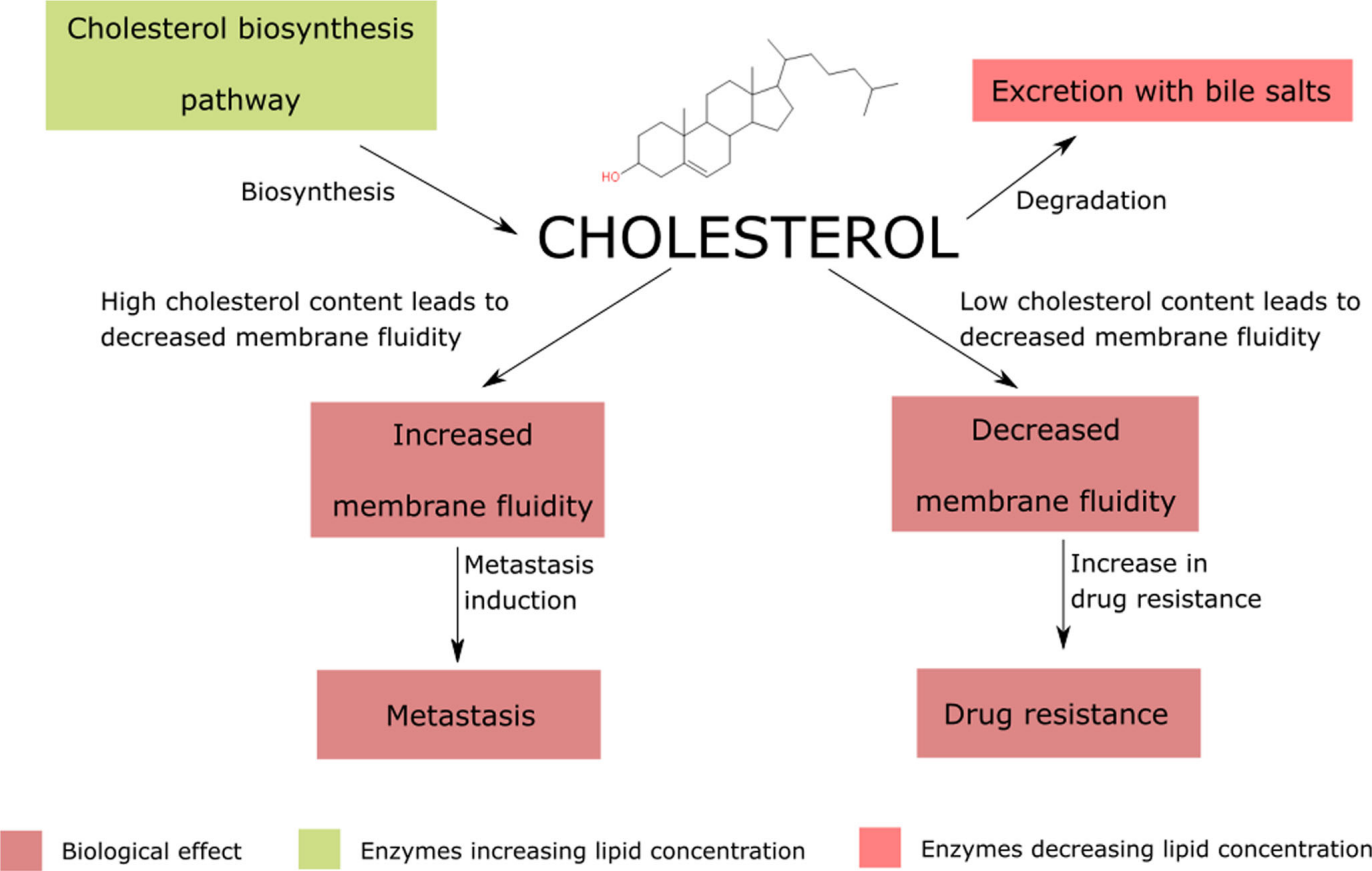
Cholesterol is the key regulator of the membrane’s fluidity. The increased membrane rigidity leads to the drug resistance by modulation of xenobiotics transporters and the physical changes in the plasmalemma, Conversely, the enhanced fluidity enables the cell to penetrate through the extracellular matrix and circulation.

Cholesterol decreases membrane fluidity under physiological conditions (*Ayee and Levitan,* 2016). The sterol is present on both sides of the membrane, however more it is abundant in the outer leaflet (*Liu et al.,* 2017a). Due to its small molecular volume and excessive hydrophobic interactions with fatty acids, cholesterol increases membrane density (*Gracià et al.,* 2010). Various cell types exhibit different levels of membrane cholesterol, for instance, Jurkat cell line cholesterol remains about 20 mol%, while breast cancer (MDA-MB-231 cell line) levels reach about 35 mol% (*Hoejholt et al.,* 2019). Cholesterol content is directly associated with cell function. Plasma membrane cholesterol is either a product of intracellular biosynthesis or a derivative of plasma lipoproteins. Consequently, any enzyme deficiency in the cholesterol metabolism pathway may result in abnormalities of membrane fluidity (Fig. 7) (*Gondré-Lewis et al.,* 2006). Due to intracellular cholesterol biosynthesis, non-motile breast cancer cells exhibit increased levels of membrane sterols (*Ehmsen et al.,* 2019). Conversely, motile Jurkat cells require lower membrane cholesterol levels to increase plasma membrane flexibility, and thus to improve the ability to infiltrate various tissues.

Cholesterol content plays a key role in membrane fluidity regulation, which modulates the resistance to the chemotherapy and metastatic properties of cancer cells. Cancer cells preparing for metastasis tend to exhibit lower membrane cholesterol levels to maximise membrane fluidity (Yang and Chen 2018; *Zhao et al.,* 2019). This feature enables a neoplastic cell to easily modulate its shape. Conversely, higher membrane cholesterol levels account for chemotherapy resistance (*Hutchinson et al.,* 2018). It has been proven that cells with higher membrane cholesterol concentration tend to exhibit greater drug resistance compared to those with lower cholesterol content (*Alves et al.,* 2016; *Peetla et al.,* 2013). This may arise from the plasma membrane sealing, by the reduction of empty spaces in the lipid bilayer. Interestingly, mitochondrial membranes stiffness seems to be involved in the resistance of neoplastic cells to death signal and the stimulation of the Warburg effect (*Ribas et al.,* 2016). Alterations in cholesterol content to enhance cell adaptation to the unfavourable environment are observed among steroidogenic cells, such as breast or ovarian carcinomas (*Chimento et al.,* 2019).

Cholesterol and sphingomyelin are involved in the formation of membrane lipid rafts (*Pike* 2004). Lipid rafts are plasma membrane regions with a highly organised lipid composition, which act as specific platforms for integral membrane proteins (IMP) concentration and stabilisation (*Simons and Ehehalt,* 2002). Flotillin and caveolin are considered as the peptide markers of the lipid rafts (*Buschiazzo et al.,* 2013). The effect of treatment could affect the structure of the raft and modulate the cancer cells, for instance, resveratrol shifts flotillin from intracellular vesicles to the raft domains in plasmalemma (*Gomes et al.,* 2019). Other research studies indicate that the reduction of plasma cholesterol content results in lipid raft destabilisation and dysfunction of the cells (*Buschiazzo et al.,* 2013). Other lipids are important in raft formation as well. In this case, phospholipid headgroups play a minor role, contrary to fatty acid residues (*Fan et al.,* 2010; *Hancock* 2006). A degree of fatty acid unsaturation and its chain length are of great importance for raft formation process (*Fan et al.,* 2010). Lipid rafts provide the environment for proteins’ oligomerization and enable interactions with other peptides (*Hancock* 2006). The high-density regions composed of cholesterol and sphingomyelin formate, constitute a proper microenvironment for proteins, which directly spans the membrane (i.e. ion channels, CD95 death receptor, T and B-cell receptors) and for proteins anchored via GPI, like alkaline phosphatase or DAF (decay-accelerating factor) (*Brown and Waneck,* 1992; *Gajate and Mollinedo,* 2015; *Zech et al.,* 2009). Rafts also supply the ions channels with cholesterol, which are essential for their proper functioning.

Since the domains are involved in a critical cell cycle processes, such as the control of cell death and survival, they are involved in carcinogenesis (*Gajate and Mollinedo,* 2015; *George and Wu,* 2012; *Groux-Degroote et al.,* 2017; *Zhuang et al.,* 2005). Molecular studies showed that the disintegration of cholesterol microdomains could be achieved by various lipid-like molecules, like erucylphosphocholine or even lysophospholipids (*Wnętrzak et al.,* 2015). The agents lead to the membrane fluidisation and destabilisation of cancer-associated markers (*Wnętrzak et al.,* 2015). Low cholesterol content results in the improper formation of rafts, defective functioning of adhesion molecules and eventually to metastases (*Buschiazzo et al.,* 2013). Indeed, tumour cells regulate the cholesterol content to allow the migration and decrease the membrane permeability for chemotherapeutics (*Mollinedo and Gajate,* 2020). For instance, melanoma migration is regulated by changes in lipid rafts (*Wang et al.,* 2013). By Src-RhoA-Rock pathway, cancer cells modulate the actin cytoskeleton, allowing intravasation and migration in the extracellular matrix (*Wang et al.,* 2013). Conversely, as far as the breast cancer cells are concerned, association to the lipid rafts restricts the interaction between CD44 and ezrin (*Donatello et al.,* 2012). In non-invasive cells, CD44 is localised in lipid rafts and ezrin aside from the non-raft regions (*Donatello et al.,* 2012). After the induction of migration, both proteins interact, leading to the metastasis (*Donatello et al.,* 2012).

### Membrane sphingolipids

Sphingolipids are heterogeneous classes of lipids, all of which contain a sphingosine backbone (*Van Meer* 2005). Sphingolipids include ceramides (sphingosine and fatty acid residue), sphingomyelins (choline or ethanolamine group esterified with a 1-phosphate group of sphingosine) cerebrosides (glucose or galactose attached to C1 of ceramide) and gangliosides (at least one sialic acid molecule attached to the sugar chain of glycolipid). Each type of the sphingolipids is discussed in the following sections.

### Ceramide and its derivatives

Under physiological conditions, ceramide content in the membrane is negligible, not exceeding 0.5 mol% (*Zech et al.,* 2009). Low concentration is a result of ceramide-mediated apoptosis induction (*Woodcock* 2006). The proapoptotic ceramide pathway begins with neutral sphingomyelinase activation by the factor associated with neutral sphingomyelinase (FAN), that is dependent on the signal from CD95 receptor death domain (DD) (*Van Blitterswijk et al.,* 2003). Ceramide, which is a product of the sphingomyelinase-catalysed reaction, induces cytochrome c release from mitochondria and consequently activates the apoptotic pathway (*Mullen and Obeid,* 2012). Increased ceramide membrane levels result in inhibition of cancer cell proliferation and proper regulation of the cell cycle (*Ogretmen* 2017). Therefore, the increased ceramide biosynthesis appears to be harmless, or even has therapeutic potential (*Woodcock* 2006). Some novel strategies of cancer therapies aim to overexpress the ceramide biosynthetic pathway enzymes and eventually lead to programmed cancer cell death (*Moro et al.,* 2019). Enzymes utilised in cancer treatment include serine C-palmitoyl transferase (CPT) - sensitising breast cancer cells to chemotherapy (*Wang et al.,* 2002), ceramide synthase 1 (CERS1) - inducing mitophagy and acute myeloid leukaemia (AML) cell death (*Dany et al.,* 2016), ceramide synthase 6 (CERS6) - modulating caspase activation in head and neck cancer (*White-Gilbertson et al.,* 2009), dihydroceramide desaturase (DES) - leading to cell cycle arrest in neuroblastoma cells (*Kraveka et al.,* 2007), acidic sphingomyelinase (ASMase) - inducing lymphoblasts apoptosis (*Santana et al.,* 1996) and neutral sphingomyelinase (NSMase) – utilised in breast cancer therapy (*Hwang et al.,* 2015).

Malfunction or overexpression of enzymes involved in the ceramide degradation pathway results in cell cycle dysregulation and apoptosis impairment, which could eventually lead to carcinogenesis (Huang et al. 2011). Increased activity of enzymes catalysing pro-malignant sphingolipids formation reactions, such as glutamate-cysteine ligase (GCL) (Li et al. 2014), spermine synthase (SMS) (*Massaro et al.,* 2017), sphingosine kinase (SphK) (*Hatoum et al.,* 2017) and ceramide kinase (CERK) (*Payne et al.,* 2014) results in the apoptosis escape and cancer progression. Excessive transport of ceramide from the endoplasmic reticulum to the Golgi apparatus is considered as pro-cancerous by separating the antiapoptotic lipid from the cell membrane (*Hanada* 2010; *Liu et al.,* 2017b).

Plasma membrane bilayer contains various amounts of sphingomyelin, oscillating between 3 mol% in Jurkat, HT-29, MDA-MB-231 and fibroblast cell lines, as well as up to 15 mol% in nerve cells (*Hoejholt et al.,* 2019; *Slotte and Ramstedt,* 2007). Sphingomyelin is found on both sides of the membrane, with a higher concentration in the outer leaflet (*Van Meer et al.,* 2008). Most lipids consist of a sphingosine chain with the oleic acid residue attached (*Hoejholt et al.,* 2019). Sphingomyelin biosynthesis involves sphinganine synthesis, further acylation to dihydroceramide and eventually dehydrogenation (*Jiang et al.,* 2009). Conversely, sphingomyelin breakdown results in ceramide release.

**Fig. 8.**
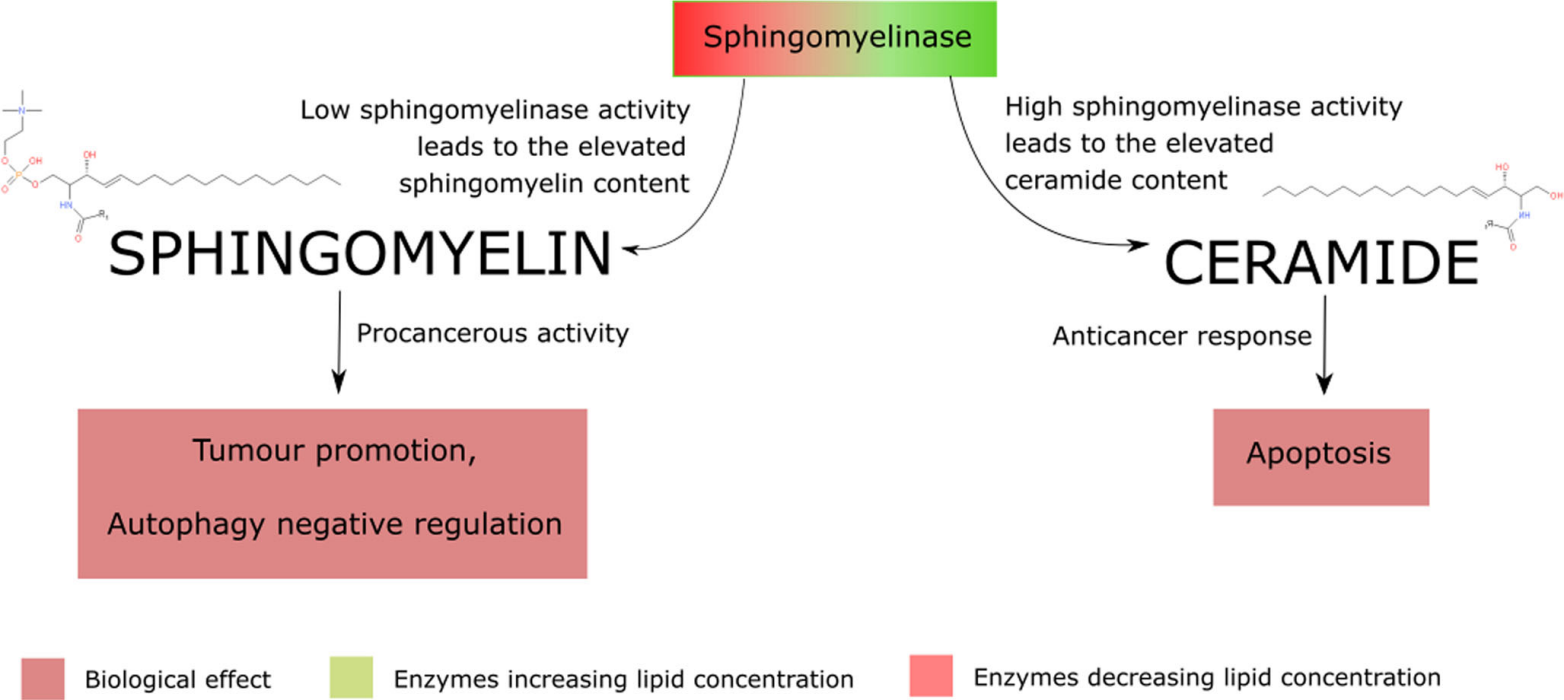
Sphingomyelin and ceramide metabolism is regulated by sphingomyelinase activity. The catalysis of choline detachment leads to cell apoptosis. However, in cancer cells, the sphingomyelinase activity is upregulated and results in the sphingomyelin extensive accumulation. The lipid enables tumor progression and autophagy negative regulation.

Sphingomyelin induces an increase in resistance to chemotherapy, by P-glycoprotein trafficking to plasma membrane lipid rafts and the increase in cell membrane integrity (*Modok et al.,* 2004; *Slotte and Ramstedt,* 2007). Besides, the increased level of sphingomyelin act as a tumour-promoting factor (*Zheng et al.,* 2019). This pro-malignant activity arises from the decrease of pro-apoptotic ceramide (*Woodcock* 2006). Moreover, sphingomyelin has been identified as a negative regulator of autophagy by impairing autophagosomes organisation, normally modulated by autophagy-related protein 9A (ATG9A) (Fig. 8) (*Corcelle-Termeau et al.,* 2016).

**Fig. 9:**
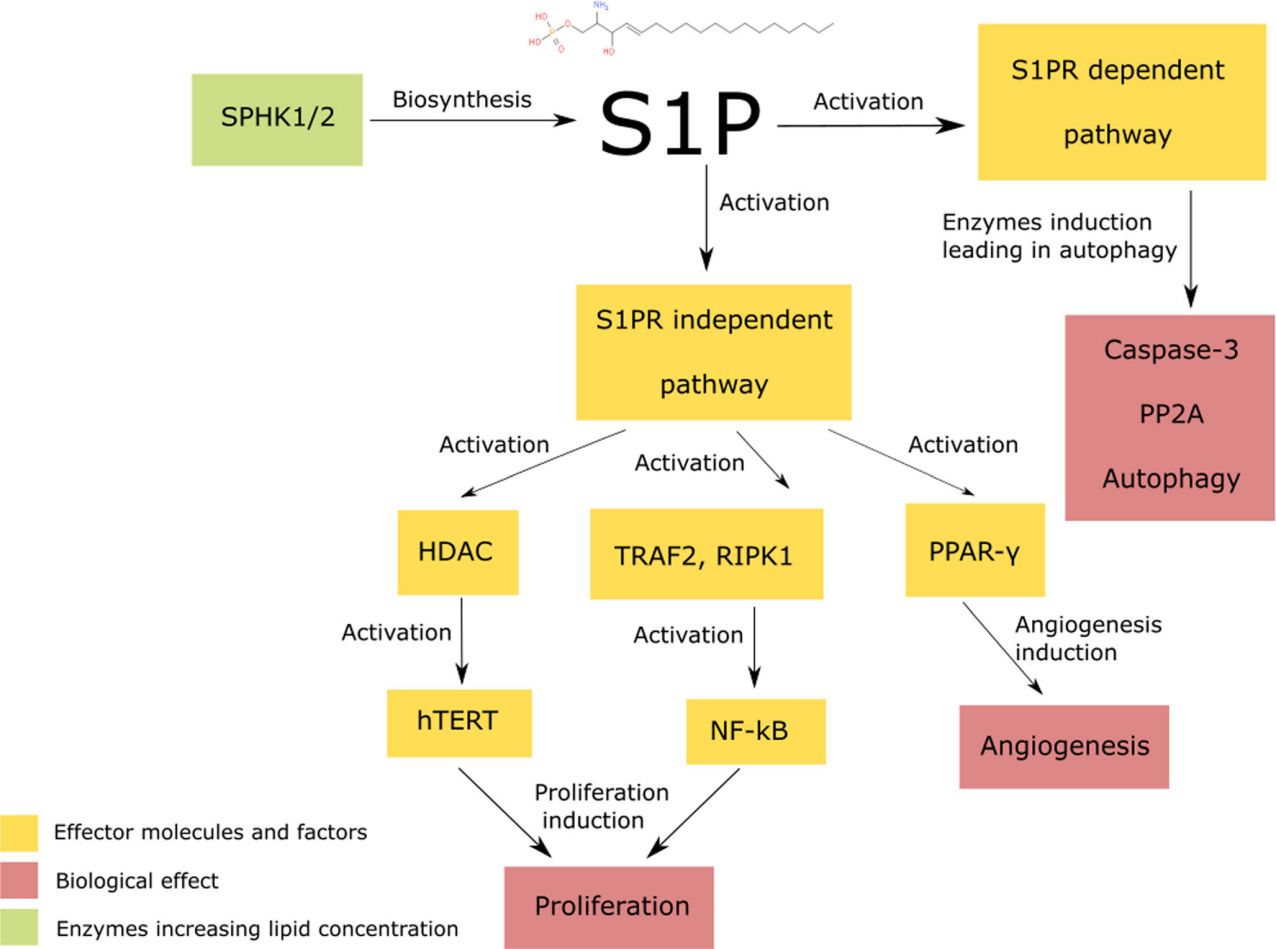
Sphingosine-1-phosphate (S1P) acts in both S1PR dependent and independent pathways. The induction of S1PR dependent pathway leads to the autophagy induction. The S1PR independent pathway activity results in increased angiogenesis and cancer proliferation; SPHK1/2 – sphingosine-1-phosphate kinase 1/2, S1PR - sphingosine-1-phosphate receptor, HDAC – histone deacetylase, hTERT – human telomerase reverse transcriptase, TRAF2 - TNF receptor-associated factor 2, RIPK1 - receptor-interacting serine/threonine-protein kinase 1, NF-kB – nuclear factor kB, PPAR-γ - peroxisome proliferator-activated receptor γ, S1PR – sphingosine-1-phosphate receptor, PP2A - protein phosphatase 2A;

Sphingosine 1-phosphate (S1P) is considered to be a tumour-promoting factor, as its elevated level promote proliferation, therapy resistance, and metastatic spread (*Guo et al.,* 2015; *Tsuchida et al.,* 2017). It is formed by sphingosine phosphorylation, catalysed by two isoforms of sphingosine kinase - intracellular (SPHK1) and nuclear (SPHK2) (*Hatoum et al.,* 2017). S1P acts either on a receptor-dependent (S1PR) or on a fully independent pathway (*Park et al.,* 2013). The independent pathway involves S1P interaction with histone deacetylase (HDAC) resulting in enhanced activity of human telomerase reverse transcriptase (hTERT), finally leading to infinite cell proliferation (*Panneer Selvam et al.,* 2015). Moreover, angiogenesis may be stimulated by the interaction of S1P with peroxisome proliferator-activated receptor-γ (PPARγ) (*Parham et al.,* 2015). Eventually, S1P accelerates cell proliferation by activating the NF- κB factor on both TNFR-associated factor 2 (TRAF2) and receptor-interacting protein 1 (RIPK1) pathways (*Alvarez et al.,* 2010; *Park et al.,* 2015). Besides, S1P- inhibits caspase-3 and protein phosphatase 2A (PP2A) as well as induces autophagy (Fig. 9) (*Watters et al.,* 2011).

### Glycosphingolipids

Glycosphingolipids could be divided into two general categories: cerebrosides with a single monosaccharide residue, and gangliosides with at least one sialic acid residue attached to the ceramide (*Yeagle* 2016). Glycolipids are localised in the outer leaflet of the plasma membrane, with the glycan chains exposed to the extracellular space (*Van Meer et al.,* 2008). Depending on the sugar residue attached, cerebrosides could be further categorised as gluco- and galactocerebrosides. A high concentration of cerebroside in the plasma membrane may act as a metastatic and antiapoptotic signal (*Liu et al.,* 2013). Cells with elevated cerebroside level tend to be more chemotherapy-resistant than those with physiological cerebroside content, due to the induction of p53 gene expression (*Hosain et al.,* 2016). The chemotherapy resistance mechanism involves direct activation of c-Src (Proto-oncogene tyrosine-protein kinase) and β-catenin signalling pathway by cell membrane cerebrosides (*Liu et al.,* 2010). This results in upregulation of multidrug resistance gene 1 (MDR1) expression as well (*Ghandadi et al.,* 2019). Cerebrosides levels rise in numerous cancer cell lines, as an effect of glucosyl- and galactosyltransferase overexpression (*Chuang et al.,* 2019; *Zhu et al.,* 2005).

In non-malignant human cells, gangliosides consist of acetyl (NeuAc) or glycol group (NeuGc) attached to the neuraminic acid by an N-glycosidic bond (*Asakawa et al.,* 2006; *Chammas et al.,* 1999). The acetyl substituent is predominant under physiological conditions (*Asakawa et al.,* 2006; *Chammas et al.,* 1999). However, cancer cells produce an excess of gangliosides that contain glycol groups (*Krengel and Bousquet,* 2014). The changes in gangliosides number and structure occur also during embryogenesis (*Breimer et al.,* 2017).

In general, a greater number of gangliosides in the lipid bilayer is associated with enhanced invasive properties of cancer (*Groux-Degroote et al.,* 2017). Studies indicate that melanoma cells exhibit significantly higher ganglioside GD3 levels in comparison to non-malignant melanocytes (*Pukel et al.,* 1982). Other neoplastic cells also express specific gangliosides, such as GD2, on the surface of leukemic T-cells (*Furukawa et al.,* 1993). Overexpression of neoplastic gangliosides also results in tumour-specific immune response inhibition via interaction with Siglec-7 protein (*Kawasaki et al.,* 2010). Moreover, cancer cells tend to release large amounts of gangliosides to the neoplastic microenvironment (*Chung et al.,* 2017). Interactions with ganglioside receptors on the surface are crucial for vascular endothelial growth factor (VEGF) signalling pathway activation, resulting in angiogenesis (*Lang et al.,* 2001). Mutations or altered expression of genes involved in ganglioside biosynthetic pathways result in ganglioside membrane levels abnormalities. Despite the great importance of gangliosides and cerebrosides in cancer development, it should be remembered that the lipids act also as antigens, and therefore their role may be much more complex (*Chung et al.,* 2017).

### Relationship between degree of unsaturation and neoplasia

Fatty acid residues may differ from one another by the degree of unsaturation, as well as the location of double bonds (*Rodwell et al.,* 2015). Bearing in mind that under physiological conditions fatty acids contain double bonds in the cis configuration, unsaturated lipids tend to increase the volume of the plasma membrane, simultaneously decreasing its density (*Ollila et al.,* 2007). Moreover, a high content of unsaturated fatty acids, makes the cell membranes more susceptible to lipid peroxidation (*Porter et al.,* 1995).

Interestingly, plasma membrane in cancer cells exhibits a different degree of unsaturation than that in non-malignant cells (*Li et al.,* 2017; *Mason et al.,* 2012; *Peck et al.,* 2016). To protect plasma membrane lipids peroxidation, cancer cells maintain a lower degree of unsaturation (*Beloribi-Djefaflia et al.,* 2016; *Peck et al.,* 2016). Moreover, a higher concentration of saturated fatty acids in the lipid bilayer results in an increased stability of lipid raft domains, resulting in greater protein binding capacity of the membrane (*Beloribi-Djefaflia et al.,* 2016).

Nevertheless, not all cancerous cells show increased saturated or monounsaturated fatty acids content. Cancer stem cells (CSCs) usually exhibit a higher degree of unsaturation as compared to non-malignant cells (*Li et al.,* 2017). Nuclear factor κB (NF-κB), directly regulates the expression of CSCs lipid desaturase, which catalyses the conversion of saturated into unsaturated fatty acids with cytochrome b5/NADH cytochrome b5 reductase electron transfer system support (*Mukherjee et al.,* 2017). Polyunsaturated fatty acids promote the expression of CSCs markers, aldehyde dehydrogenase (ALDH) and CD133 among cancer cells with CSCs phenotype (*Yi et al.,* 2018).

### Lipid membranes as a therapeutic target

Nowadays, the lipid content of the cancer cell is pharmacologically modulated, as a part of clinical management (*Luo et al.,* 2018). The regulation of lipid metabolism is especially effective among carcinomas with highly altered lipid content. The therapy does not only concern the plasma membrane lipids, but also the intracellular membranes, such as the endoplasmic reticulum or Golgi apparatus (*Shyu et al.,* 2018). Drugs used in lipid-targeting therapies could be divided into two groups.

The first group consists of drugs that specifically affect the lipid or its metabolism (*Cheng et al.,* 2016; *Tan et al.,* 2017). Due to the fact that the targeted lipids build all the cells in the body, the therapy has to be very specific affecting only a certain lipid and carried out in elevated concentrations range (*Tan et al.,* 2017). In this field researchers particularly focus on the design and production of very specific proteins or small-molecule compounds to omit the severe systemic complications of the therapies (Srivatsav et al. 2018; *Bernardes and Fialho,* 2018; *Yan Lim and Yee Kwan,* 2018). As discussed before, the therapy results in the changes in the cell membranes properties, resulting in carcinogenesis, metastases and drug resistance decrease (*Frimpong* 2019; *Zalba and ten Hagen,* 2017). To maintain the desired specificity during chemotherapy, it is required to use drugs that target certain lipid metabolism (*Cheng et al.,* 2016; *Tan et al.,* 2017). Such an approach was effectively applied for carcinomas, lipid metabolism dysregulation diseases, such as ovarian cancer, sarcomas or even pancreatic endocrine tumours (*Manara et al.,* 2010; *Soler et al.,* 2016; *Xie et al.,* 2017).

The second group of lipid-lowering drugs, which have been proved to have anticancer properties are statins (i.e. Lovastatin) (*Peng et al.,* 2017). The mechanism statins action involves the disruption of cholesterol biosynthesis, decrease of cell proliferation and survival (*Martirosyan et al.,* 2010). From the histological point of view, the changes being a consequence of the therapy include a lack of lipid droplets in the affected cells (*Gbelcová et al.,* 2013). This approach was effectively used among carcinomas with high membrane cholesterol content, such as breast cancer (*Borgquist et al.,* 2018). Interestingly, clinical metanalysis of cancer incidence among patients treated with statins showed an increased probability of high-grade prostate cancer diagnosis (*Nordström et al.,* 2015). Moreover, Shepherd et al. proved that patients treated with pravastatin were more frequently diagnosed with cancer in comparison to the placebo-treated group (*Shepherd et al.,* 2002). Aside from the pro-cancerous effects, statin use in chemotherapy meets obstacles, such as the use of high doses (500 times higher than standard hypercholesterolemia treatment) of the drug (*Dulak and Józkowicz,* 2005). The high anticancer concentration of the drug leads to the statin-induced rhabdomyolysis (*Hu et al.,* 2012). Therefore, statins could be used as adjuvant therapy for cancer treatment in the mevalonate pathway targeting (*Mo et al.,* 2019). The blockage of mevalonic acid synthesis leads to the deactivation of isoprenoid-composed G-protein-coupled proteins, such as Ras, Rho and Rac (*Vallianou et al.,* 2014). Additionally, statins could enhance the effects of the immune response towards cancer by increasing the antigen presentation by dendritic cells (*Bird* 2018).

The most current management aims to block the uptake, intracellular lipolysis and utilisation of lipids (*Diao and Lin,* 2019). This approach was introduced, when researchers found that cancer cells are characterised by high energy demand for proliferation (*Long et al.,* 2018). Overexpression of lipid uptake receptors (like CD36) in the tumour came out to be an effective target to suppress cancer growth and progression (*Pascual et al.,* 2017). A distinction of the lipid composition of the cell membrane in various types of cancer cells might be useful in the planning of anticancer strategy. The exemplary profiles according to the available data are presented in Table 1.

**Table 1:** Cell membrane lipid composition based on the lipidomic studies of the cell membrane of various cell lines

**Lipid [mol%]**	**HT-29**	**MDA-MB231**	**HDF-n**	**Jurkat**
PA	0.5	0.5	0.5	0.3
PI	4	4	4	12.5
PG	0.5	0.5	0.5	0.3
PS	4	4	4	7.5
PE	2	4	8	14
PC	9.5	13	20.25	40
PE eter	7.25	10.5	7.75	3
PC eter	16.75	1.5	0.25	3.4
SM	2.5	8.3	5.83	4
DAG	45.8	51.7	45.8	0.5
Chol				23
HexCer				0.1
diHexCer				0.01

HT-29 – colon cancer, MDA-MB231 – breast cancer, HDF-n - human dermal fibroblasts, Jurkat – T-cell leukaemia. mol% values correspond to the approximate values of each lipid. PA – phosphatidic acid, PI - phosphatidylinositol, PG - phosphatidylglycerol, PS - phosphatidylserine, PE - phosphatidylethanolamine, PC - phosphatidylcholine, SM - sphingomyelin, DAG - diacylglycerol, Chol - cholesterol, HexCer - hexosylceramides, diHexCer – dihexosylceramides

Presented data, show an increased PI level in Jurkat cell line. The extent of PI leads to cancer cells proliferation and changes in G-protein mediated signalling pathways. The hypothetical therapy approach would include Ras or Rho inhibitors. Indeed, molecular and clinical data, prove the high contribution of Ras in leukaemia (*Kindler et al.,* 2008; *Pasmant et al.,* 2015). Moreover, Ras inhibitor - farnesyl thyosalicylic acid was successfully used to sensitise the T-cell leukemic cells to apoptosis signal (*Stoppa et al.,* 2012). According to the data presented in Table 1, PC, PS and PE depleting agents could potentially be useful to target leukaemia cells. In the case of PS, fingolimod (FTY720) was successfully used to induce phosphatidylserine externalisation, thus leading to cell death (*Young et al.,* 2019). No clinical data could be found in the field of PC or PE depletion, however, in vitro research showed the potency of trans,trans-farnesol (PC uptake inhibitor) in death induction in leukemic cells (*Melnykovych et al.,* 1992). Conversely, the excess of cholesterol was proved to be a potential therapeutic target in breast cancer treatment (*Li et al.,* 2006). On the other hand, its depletion together with metformin and methyl-β-cyclodextrin resulted in the reduced migration and cell death induction (*Guerra et al.,* 2016; *Sharma et al.,* 2019).

Inhibition of lipid biosynthesis and its uptake provides the physicians with a complete set of tools and possibilities to modulate the lipid composition of the cancer cell.

## Summary

Lipid profile may be considered as a crucial prognostic factor in cancer patients. Quantitative changes in lipids are observed in all neoplastic cells as compared to non-malignant cells. Therefore, some lipids (e.g. phosphatidylcholine) may be considered as tumour markers in certain malignancies. Alternations in the main groups of the membrane lipids are mostly associated with activation and inactivation of cellular signalling pathways. In some cases, the changes result in the metabolic reprogramming of tumour cells. On the other hand , alterations made to fatty acids chains length and their degree of unsaturation, change the physical properties of cell membrane - either increasing its fluidity or stabilising membrane proteins in the lipid rafts. Additionally, a significant role in cancer therapy resistance mechanisms and metastases is played by cholesterol which is responsible for membrane fluidity regulation..

There is still much to be discovered on the lipid composition of the cancer cell membrane, however, the current knowledge can already be successfully utilised in cancer treatment.
